# Charismatic and Visionary Leaders

**DOI:** 10.1523/ENEURO.0125-21.2021

**Published:** 2021-04-27

**Authors:** Eve Marder

**Affiliations:** Volen Center and Biology Department, Brandeis University, Waltham, MA 02454

**Keywords:** computational neuroscience, failure of averaging

Charismatic leaders play fascinating roles in society, for good and for ill. In the United States, we are aware of the power of Martin Luther King and John Lewis in the fight for equal rights, and every country will have its heroes in its struggles for freedom. And, we are equally aware of the impact of despots who have led to terrible miscarriages of justice. We are less accustomed to think of the roles of charismatic leaders in our scientific communities, as we prefer to believe that we all “follow the science” rather than the scientist! Nonetheless, my eyes were opened to the impact of personal charisma in science at the Society for Neuroscience Meeting in 1980. I was chatting with a very fine membrane biophysicist when Eric Kandel walked by and my colleague said, “Kandel is my hero.” I was surprised, as the work my friend did was detailed characterization of membrane currents, far from the grand sweep of Kandel’s work. And when I asked my friend why he revered Kandel, he said that he didn’t dare to dream to big picture questions but restricted himself to elucidating the details of cellular mechanisms precisely because of his own lack of visionary courage. My friend valued Kandel, not for the details of his experiments, but for articulating that someday it would be possible to establish the molecular and cellular bases of learning and memory. I learned from this conversation that scientists add value or are destructive to our collective enterprise in disparate ways.

Henry Markram is one of these fascinating individuals who dared to set himself and our community the most ambitious of goals. I first met Markram early in his career when he was doing breathtaking and virtuoso work in brain slices, first as a postdoc and then as a beginning faculty member. Years later, I was in the audience for Henry Markram’s talk at the 2012 FENS meeting in Barcelona.

I experienced Henry’s Barcelona talk as I imagine some people experience an evangelical prayer meeting. The screen was floor to ceiling, and filled with extraordinarily stunning images of neurons. Markram, in a very low-key voice, articulated the goal of building a model that would lead to enlightenment (an understanding of how the brain works). I sat there and was captured by his spell, as the beauty of the biological images made it easy to fall in line with his vision. At the same time, my logical self asserted that the scientific program he was advocating and implementing could never lead to enlightenment because it was constructed on a series of questionable premises. Consequently, I was deeply frustrated, as Markram’s program was both awe-inspiringly ambitious, meticulous, and on the surface elegant but combined with many systematic oversimplications that, for me, invalidated the enterprise.

What were my scientific objections at that time as I remember them? First, Markram’s initial goal was to build a model of a cortical column and to validate it using slice data. I understood the benefits of trying to capture the dynamics of a column-sized piece of brain, but columns in the brain do not function alone, and neurons within one column receive and send information to many other brain areas. So what kind of output could one measure from a cortical column in a slice, or for that matter in the brain to constrain the model? Or even to know if the model was producing a sensible output? Second, Markram started his career as a cellular and synaptic patch biophysicist (Markram and Tsodyks, 1996; Markram et al., 1997, 2004). By 2012, Markram’s group was collecting data that were rich in detail and complexity in large quantities. Then, the proposed process was to average these data and build the model from mean data. By this point, we and others had come to understand the dangers of using mean data (Golowasch et al., 2002), as the mean of a population can have properties quite different from the individual measurements that were used to calculate the mean. I was particularly sensitive to this, as we had been systematically measuring the variability in channel gene expression in the same identified neurons across animals and were seeing considerable variability (Schulz et al., 2006, 2007). Third, Markram was not proposing to tune the model to a desired outcome, but at the time argued that because of the realism of the biological data, that the model would automatically work. This struck me as magical thinking that depended on not having a well-defined behavioral output. Fourth, regardless of how elegant work in brain slices may be, the properties of those neurons are almost certainly different from those the same neurons would show in the intact brain. This isn’t a criticism of the utility of elegant biophysics done in slice for understanding the basic biology of neurons and synapses, but just of their limitations as we try to understand the dynamics of the working brain.

Ironically, Markram’s proposed workflow bore witness to his deep respect for the richness in the biology and then essentially invalidated it by averaging it away. In so doing, the program de facto failed to confront one of the deepest conundrums that we face in understanding the brain: how do we understand how brain dynamics depend on the details of brain connectivity and individual neuron dynamics? In all fairness to Markram and his colleagues, there was then no simple solution to this problem. And the problem is still at the core of many conversations about the use, or lack thereof, of the increasingly large datasets we are today collecting. Indeed, much of the work done today under the auspices of the United States Brain Initiative or the Allen Institute (Jorgenson et al., 2015; Abbott et al., 2020; Gouwens et al., 2020; Miller et al., 2020; Yuste et al., 2020) faces the same intellectual challenges of trying to understand how to tackle the computational implications of large numbers of neurons, each of which is individually complex, with, to greater and lesser degrees, variable properties.

So in 2012, I walked out of that room sad. I was sad that the extraordinary beauty of neuroscience was compromised by, for me, a flawed vision. That said, I was not surprised by those who were disciples of the vision. I always felt and understood the draw of the beauty and that there would be those for whom the intrinsic truth of the beauty would outweigh the flaws of the program. And I knew that were always going to be those whose very being would be offended by the idea of a monolithic and single-minded collective project.

However, it is important to remember and acknowledge that a truly ambitious and global proposed enterprise will have inadvertent results over and above those intended. Indeed, the conversations and debates catalyzed by the Blue Brain and the Human Brain Project forced the entire field of neuroscience to engage in serious debates about what can and cannot be done, how resources should be spent, and what success in understanding the brain might look like. The European Human Brain Project had substantial impacts on the United States Brain Initiatives, and other large international projects. Consequently, a major piece of Markram’s success, albeit underappreciated as it may be, was in capturing the attention of so many people to argue about what the entire community should or should not be doing. If he were less charismatic and had his initial vision been less astronomically ambitious, the intensity and the fervor of the ensuing conversations might not have occurred, and the entire field would be the poorer for the lack of those debates.

When I watched Noah Hutton’s film *In Silico*, I felt that the film did not really show us the extraordinary beauty of the neuroscience but focused rather on the failure of the early vision. It is an outstanding chronicle of the filmmaker’s maturation and interesting as it spans ten years of his life and of neuroscience. Almost 10 years after the FENS meeting in 2012, I feel today it is important to celebrate the beauty and the scope of some of the science that was done (Ranjan et al., 2019) as well as to acknowledge the hubris of the attempt and the extent of its apparent failures. The hubris and the beauty are two sides of the same coin. Without the extraordinary beauty of the images and much of the spell-binding nature of the data collected, the hubris could not have won the hearts and minds of any, much less many. As I grow older, I cherish more our charismatic leaders who search for answers to the secrets of the universe and the brain, knowing how far away that understanding remains. At the same time, I feel it more and more important for us to hold each other accountable for the veracity of our claims. It is just very hard to climb to the stars with only a fragile ladder that only reaches part of the distance.

## Synthesis

Reviewing Editor: Christophe Bernard, INSERM & Institut de Neurosciences des Systèmes Decisions are customarily a result of the Reviewing Editor and the peer reviewers coming together and discussing their recommendations until a consensus is reached. When revisions are invited, a fact-based synthesis statement explaining their decision and outlining what is needed to prepare a revision will be listed below. The following reviewer(s) agreed to reveal their identity: NONE.

N/a
